# Composition and Biosynthesis of Scent Compounds from Sterile Flowers of an Ornamental Plant *Clematis florida* cv. ‘Kaiser’

**DOI:** 10.3390/molecules25071711

**Published:** 2020-04-08

**Authors:** Yifan Jiang, Renjuan Qian, Wanbo Zhang, Guo Wei, Xiaohua Ma, Jian Zheng, Tobias G. Köllner, Feng Chen

**Affiliations:** 1 College of Horticulture, Nanjing Agricultural University, Nanjing 210095, China; 2017204038@njau.edu.cn; 2 Zhejiang Institute of Subtropical Crops, 334 Xueshan Road, Wenzhou 325005, China; qrj7@163.com (R.Q.); maxiaohua1120@126.com (X.M.); zjyzs@126.com (J.Z.); 3 Department of Plant Sciences, University of Tennessee, Knoxville, TN 37996, USA; gwei@utk.edu; 4 Department of Biochemistry, Max Planck Institute for Chemical Ecology, D-07745 Jena, Germany; koellner@ice.mpg.de

**Keywords:** *Clematis*, basal dicot, floral scent, terpene synthase, monoterpenes, linalool, *TPS-g* subfamily, spatial specificity

## Abstract

*Clematisflorida* is a popular ornamental vine species known for diverse colors and shapes of its flowers but not for scent. Here we investigated the composition and biosynthesis of floral scent in ‘Kaiser’, a fragrant cultivar of *C. florida* that has sterile flowers. Volatile profiling revealed that flowers of ‘Kaiser’ emit more than 20 compounds, with monoterpenes being most abundant. Among the three floral organs, namely sepals, transformed-petals, and ovaries, ovaries had the highest rates of total volatile emission. To determine the molecular mechanism underlying floral scent biosynthesis in ‘Kaiser’, we sequenced a flower transcriptome and searched the transcriptome for terpene synthase genes (*TPSs*), which are key genes for terpene biosynthesis. Among the *TPS* genes identified, three were putative intact full-length genes and were designated *CfTPS1*, *CfTPS2*, and *CfTPS3*. Phylogenetic analysis placed *CfTPS1*, *CfTPS2*, and *CfTPS3* to the *TPS-g*, *TPS-b*, and *TPS-a* subfamily, respectively. Through in vitro enzyme assays with *Escherichia coli*-expressed recombinant proteins, both *CfTPS1* and *CfTPS2* were demonstrated to catalyze the conversion of geranyl diphosphate to linalool, the most abundant constituent of *C. florida* floral scent. In addition, *CfTPS1* and *CfTPS2* produced the sesquiterpene nerolidol from (*E,E*)-farnesyl diphosphate. *CfTPS3* showed sesquiterpene synthase activity and produced multiple products *in vitro*. All three *CfTPS* genes showed higher levels of expression in sepals than those in transformed-petals and ovaries. Our results show that despite being sterile, the flowers of ‘Kaiser’ have normal mechanisms for floral scent biosynthesis that make the flowers fragrant.

## 1. Introduction

Among the most popular perennial climbing ornamental species is *Clematis florida*, which belongs to the Ranunculaceae family. A large number of cultivars have been developed for *C. florida*. They are widely used in landscaping and floriculture as garden or potted plants [1]. The predominant morphological features of the hermaphrodite flower of *C. flrodia* are their diverse colors and shapes. Aside from ornamental applications, the roots and rhizomes of *C. florida* have been used as anti-inflammatory, anti-rheumatism, and analgesic agents [2] due to the richness in secondary metabolites, including triterpene saponins, alkaloids, flavonoids, lignans, coumarins, macrocyclic compounds, and phenolic glycosides [3,4,5,6,7,8,9,10]. Among the traits that make *C. florida* a popular ornamental plant, the floral scent is usually not one of them. Therefore, new varieties with fragrant flowers in addition to other attractive horticultural traits are important targets for new breeding programs of *C. florida*. Although some cultivars have fragrant flowers [1], little is known about the composition and biosynthesis of floral scent in *C. florida*.

In contrast to the scarcity of information on the biology of floral volatiles of *C. florida*, much has been learned about the general topic of chemistry, biosynthesis, and biological functions of floral scent. A large number of floral scent compounds (more than 1700) have been identified [11]. Despite this large number, most floral volatiles belong to several major chemical classes, including terpenoids, phenylpropanoids/benzenoids, fatty acid derivatives, and nitrogenous compounds [12,13]. The general biochemical pathways leading to the different classes of volatiles are relatively well understood, and several key protein families such as the terpene synthase (*TPS*) family, *BAHD* acyltransferase family, and small molecule methyltransferase families have been described to play important roles in the formation of floral volatiles [14,15,16,17]. In many of these studies, the successful identification of scent-producing genes was based on the integration of metabolomic and transcriptomic data [18,19,20]. *TPS*s involved in the biosynthesis of floral volatile terpenes, particularly monoterpenes (C_10_) and sesquiterpenes (C_15_), belong to *TPS-a*, *TPS-b*, and *TPS-g* subfamilies [18,19,20,21,22,23]. Floral volatiles emitted from angiosperm plants possess multifaceted functions particularly for attracting pollinators and defending against florivores [11].

In this study, we chose the cultivar ‘Kaiser’ as a model plant to investigate the biology of floral volatiles of *C. florida*. ‘Kaiser’ has a particularly high ornamental value in both the visual and olfactory sense with the combination of flower type, color, and floral fragrance. Being different with many other cultivars, the flowers of ‘Kaiser’ are sterile due to the unique flower structure, including the petal-like sepals (calyces), transformed petal (derived from stamen), and pistil [1]. Here we present a detailed investigation on chemical composition and biosynthesis of floral volatiles of ‘Kaiser’. The new knowledge will not only provide a good understanding of floral scent biology of ‘Kaiser’, but also lay a foundation for developing new cultivars of *C. florida* with fragrant flowers.

## 2. Results

### 2.1. Chemical Composition of Floral Volatiles of ‘Kaiser’

**Figure 1:**
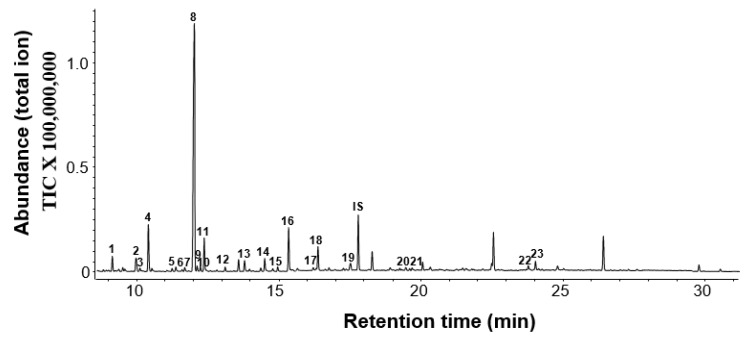
Gas chromatograph (GC) profile of floral volatile emission in *C. florida* ‘Kaiser’ at the full blooming stage. Separation of volatile compounds was performed on an Agilent HP 5 MS capillary column. Compounds identified from floral volatile blend are labeled with 1–23. (**1**) 2-Hydroxyethyl methacrylate, (**2**) Limonene, (**3**) Benzyl alcohol, (**4**) 2-Phenylacetaldehyde, (**5**) *trans*-Linalool oxide, (**6**) 3-Carene, (**7**) Linalool, (**8**) 5-Caranol, (**9**) Nonanal, (**10**) 6-Ethenyldihydro-2,2,6-trimethyl-2*H*-pyran-3(4*H*)-one, (**11**) Phenylethyl alcohol, (**12**) Benzyl cyanide, (**13**) 1,4-Dimethoxybenzene (**14**) 2-(2-Butoxyethoxy)ethanol), (**15**) Decanal, (**16**) 2-Phenoxyethanol, (**17**) Phenylacetaldoxime, (**18**) 2-Phenylethylacetate, (**19**) 2-Phenylnitroethane, (**20**) Tetradecane, (**21**) 1,3,5-Trimethoxybenzene, (**22**) 2,6,10,15-Tetramethylheptadecane, (**23**) Nerolidol. IS represents the internal standard.

Combining headspace collection with gas chromatography–mass spectrometry (GC-MS) analysis, the fully-opened flowers of ‘Kaiser’ were shown to emit more than 20 volatile compounds including seven terpenoids, eight benzenoids, five fatty acid-related compounds, and three nitrogenous volatiles. Terpenoids were the predominant class, accounting for 66.9% of the total emission (Figure 1; Table 1). Of the six monoterpenoids limonene, carene, *trans*-linalool oxide, 5-caranol, linalool, and 6-ethenyldihydro-2,2,6-trimethyl-2*H*-pyran-3(4*H*)-one found in ’Kaiser’, linalool accounted for 59.3% of the total emission (Figure 1; Table 1).

Benzenoids and fatty acid-derived compounds accounted for 28.1% and 2.4% of the total emissions, respectively. Benzyl cyanide, phenylacetaldoxime, and phenylnitroethane are aromatic nitrogen-containing compounds and contributed 2.7% to the total emission.

### 2.2. Spatial Specificity of Volatile Emission in Different Floral Organs from ‘Kaiser’

**Figure 2:**
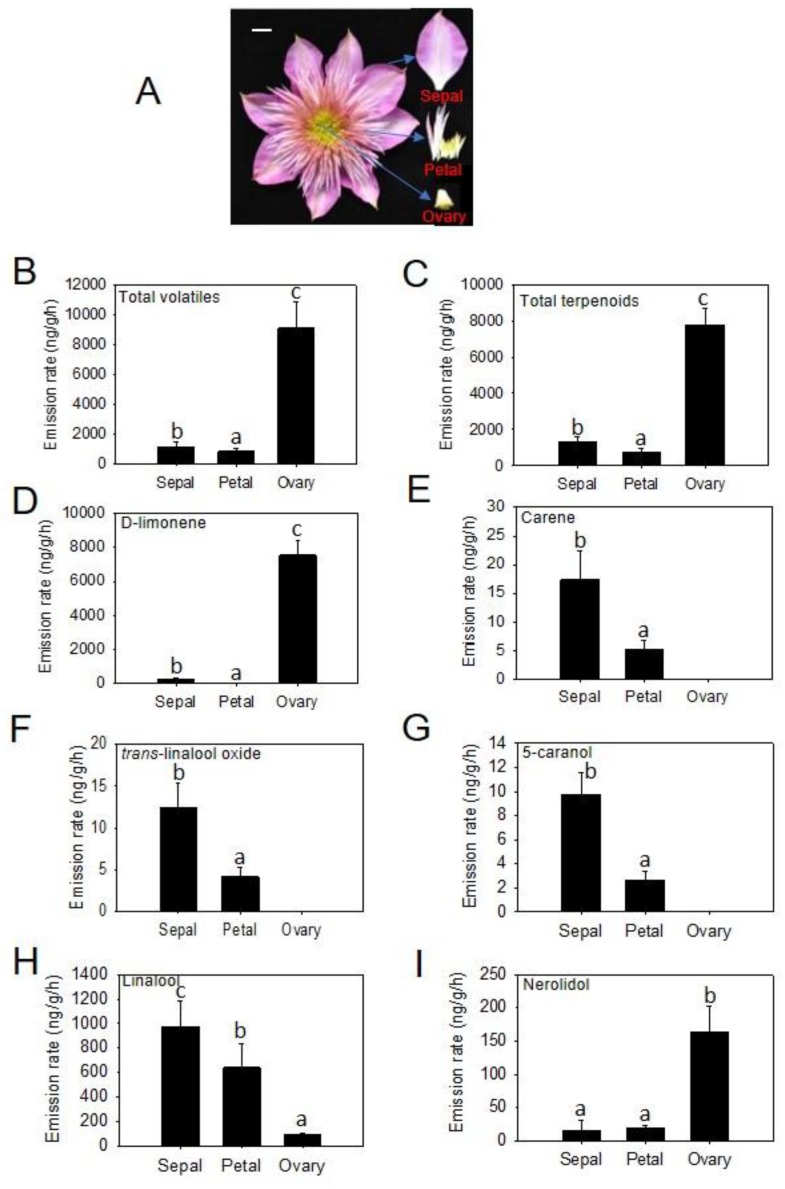
Volatile emission from different parts of *C. florida* ‘Kaiser’ flowers. Representative images of the intact flower and three detached organs of *C. florida* ‘Kaiser’ (**A**). Emission rate of total volatiles (**B**) and total terpenoids (**C**) from three detached organs in *C. florida* ‘Kaiser’. Individual terpenoids from three detached organs in *C. florida* ‘Kaiser’: limonene (**D**), carene (**E**), *trans*-linalool oxide (**F**), 5-caranol (**G**), linalool (**H**), and nerolidol (**I**). Data were obtained through semi-quantification and are presented as mean ± SE (*n* = 3). Different letters in **B**–**G** denote statistically significant differences among the means according to ANOVA analysis (*p* < 0.05).

To further investigate the patterns of floral volatile emission, the sterile flower of ‘Kaiser’ was divided into sepals, transformed-petals, and ovaries, which were subjected to headspace collection separately. 17, 10, and 15 volatile compounds were identified from sepals, transformed-petals, and ovaries, respectively (Figure 2).

Ovaries showed the highest rates of total emission (8936.55 ± 1703.16 ng h^−1^g^−1^) among the three tissues. The emission rate of total volatiles from ovaries was about six times higher than that from sepals and 11 times higher than that from transformed-petals (Figure 2B). Among the three tissues, ovaries also showed the highest rates of emission for individual chemical classes: 7793.14 ± 899.32 ng h^−1^g−^1^ for terpenoids, (Figure 2C), 710.08 ± 121.64 ng h^−1^g^−1^ for benzenoids, 224.71 ± 37.45 ng h^−1^g^−1^ for fatty acid derivatives, and 177.26 ± 23.08 ng h^−1^g^−1^ for nitrogenous volatiles. As for individual terpenes, limonene (7532.29 ± 839.18 ng h^−1^g^−1^) and nerolidol (163.52 ± 38.95 ng h^−1^g^−1^) exhibited the highest rates of emission from ovaries (Figure 2D,I). In contrast, carene, trans-linalool oxide, 5-caranol, and linalool showed the highest rates of emission from sepals (Figure 2E–H).

### 2.3. Transcriptome Sequencing and Annotation of Assembled Unigenes

To understand the molecular basis of floral volatile biosynthesis in ‘Kaiser’ flowers, a RNA-Seq library using the RNA samples of intact flowers of ‘Kaiser” at full blooming stage was constructed and sequenced. Approximately 65.93 M raw reads were obtained, and 61.72 M high-quality clean reads remained after filtering. A total of 32,048 unigenes were obtained with a maximum length of 11,031 bp, minimum length of 297 bp, and an N50 length of 1323 bp (Figure S1; Table S1).

To determine the putative functions of unigenes, the transcriptome was annotated using protein functions, pathways, euKaryotic Ortholog Groups (KOG) functions, and Gene Ontology (GO) annotations. The unigenes were aligned using the BLASTx program with an e-value threshold of 10^−5^ to the Nr (NCBI non-redundant protein), the Swiss-Prot protein, and the KEGG (Kyoto Encyclopedia of Genes and Genomes), KOG databases and GO database for which the percentages of the annotated unigenes were 77.82%, 57.68%, 59.23%, 60.62%, and 50.39%, respectively (Table S2). A total of 18,981 unigenes were annotated in KEGG pathways and 10,490 unigenes were assigned to “Metabolites” group (Figure S2A). 271 unigenes were involved into the metabolism of terpenoids and polyketids, accounting for 1.42% of the total unigenes. The GO functional annotations of the unigenes were classified into three categories (Molecular Function, Cellular Component, and Biological Process) and 39 subcategories (Figure S2B). There were 1190 unigenes assigned to subcategories “metabolic process”, accounting for 7.37% of the total unigenes (Figure S2B).

**Figure 3:**
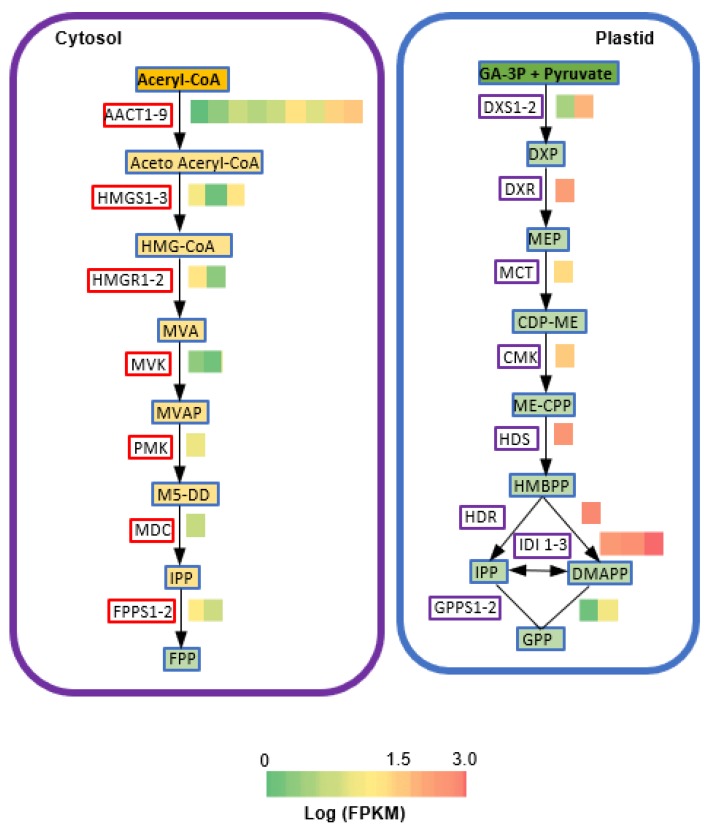
Representative terpenoid biosynthesis pathways with cognate heat maps for transcript levels of genes from *C. florida* ‘Kaiser’ transcriptome data with substrates and products. Green/red color-coded heat maps represent relative transcript level calculated with fragments per kilobase of transcripts per million mapped fragments (FPKM). Abbreviations of enzymes: *AACT*, acetyl-CoA C-acetyltransferase; *HMGS*, hydroxylmethylglutaryl-CoA synthase; *HMGR*, hydroxymethylglutaryl-CoA reductase (NADPH); *MVK*, mevalonate kinase; *PMK*, 5-phospho-mevalonate kinase, *MDC*, mevalonate diphosphate decarboxylase; *FPPS*, farnesyl pyrophos-phate synthase; *DXS*, 1-deoxy-d-xylulose-5-phosphate synthase; *DXR*, 1-deoxy-d-xylulose-5-phos-phate reductoisomerase; *MCT*, 2-C-methyl-d-erythritol 4-phosphate cytidylyltransferase; *CMK*, 4-diphosphocytidyl-2-C-methyl-d-erythritol kinase; *HDS*, (*E*)-4-hydroxy-3-methylbut-2- enyl diphosphate synthase; *HDR*, 4-hydroxy-3-methylbut-2-enyl diphosphate reductase; *IDI*, isopentenyl-diphosphate delta-isomerase; *GPPS*, geranyl diphosphate synthase. Compound abbreviations: HMG-CoA, 3-hydroxy-3-methylglutaryl-CoA; MVA, mevalonate; MVAP, 5-phosphomevalonate; M5-DD, 5-pyrophosphomevalonate; FPP, (*E,E*)-farnesyl pyrophosphate; DXP, 1-deoxy-d-xylulose 5-phosphate; MEP, 2-C-methyl-d-erythritol 4-phosphate; CDP-ME, 2-C-Methyl-d-erythritol-2,4-cyclodiphosphate; ME-CPP, 2-C-methyl-d-erythritol-2,4-cyclodiphosphate; HMBPP, 1-hydroxy-2-methyl-2-(*E*)-butenyl-4-diphosphate; IPP, isopentenyl pyrophosphate; DMAPP, dimethyallyl pyrophosphate; GPP, geranyl pyrophosphate.

Given terpenoids being the dominant floral volatile compounds of ‘Kaiser’, next, we analyzed terpenoid biosynthesis pathways in the transcriptome. There are two general biochemical pathways responsible for the formation of terpenoids in plants. One is the cytosol-localized mevalonate (MVA) pathway. The other is the plastid-localized methyl-erythritol phosphate (MEP) pathway. Both pathways lead to the formation of the C5 precursors isopentenyl pyrophosphate (IPP) and dimethylallyl pyrophosphate (DMAPP). C10 precursor geranyl pyrophosphate (GPP) and C15 precursor (*E,E*)-farnesyl pyrophosphate (FPP) are formed through the condensation of IPP and DMAPP. For the MEP pathway, multiple genes were detected for 1-deoxy-d-xylulose-5-phosphate synthase (*DXS*), isopentenyl-diphosphate delta-isomerase (*IDI*), and geranylgeranyl diphosphate synthase (*GPPS*). In contrast, a single copy gene was detected for 1-deoxy-d-xylulose-5-phosphate reductoisomerase (*DXR*), 2-C-methyl-d-erythritol 4-phosphate cytidylyltransferase (*MCT*), 4-diphosphocytidyl-2-C-methyl-d-erythritol kinase (*CMK*), and 4-hydroxy-3-methylbut-2-enyl diphosphate reductase (*HDS*). For the MVA pathway, multiple copies of genes were detected for acetyl-CoA C-acetyltransferase (*AACT*), hydroxylmethylglutaryl-CoA synthase (*HMGS*), hydroxymethylglutaryl-CoA reductase (*HMGR*)*,* mevalonate kinase (*MVK*), and farnesyl pyrophosphate synthase (*FPPS*). In contrast, a single copy gene was detected for 5-phosphomevalonate kinase (*PMK*) and mevalonate diphosphate decarboxylase (*MDC*), (Figure 3).

### 2.4. Identification of CfTPS Genes

Next, the transcriptome of ‘Kaiser’ flowers was specifically searched for *TPS* genes. Three putative full-length intact *TPS* genes were identified. They were designated *CfTPS1*, *CfTPS2,* and *CfTPS3*. The proteins they encode are 554 (*CfTPS1*), 586 (*CfTPS2*), and 591 (*CfTPS3*) amino acids in length. A sequence alignment revealed that all of the *CfTPS* proteins contained the conserved ‘DD_XX_D’ and ‘NSE/DTE’ motifs that are essential for the binding of the co-factors Mg^2+^ or Mn^2+^ to catalyze terpene biosynthesis [19].

**Figure 4:**
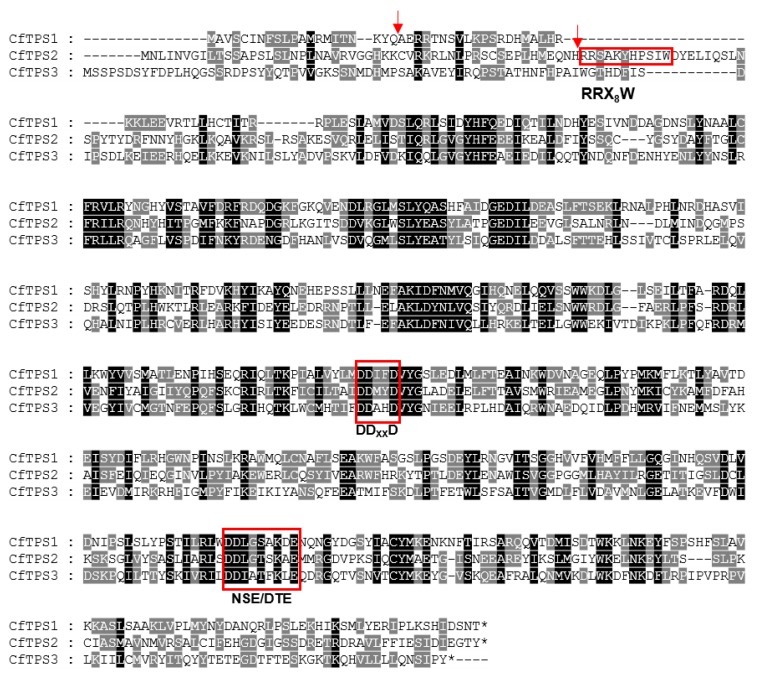
Alignment of the amino acid sequences of *CfTPS1*, *CfTPS2*, and *CfTPS3*. Amino acids identical in all three proteins are shaded in black and those identical in two proteins are shaded in gray. The three highly conserved motifs are labeled RR-(x)8-W, DDxxD, and (N,D)Dxx(S,T)xxxE, respectively. Arrows indicate putative cleavage sites of transit peptides.

**Figure 5:**
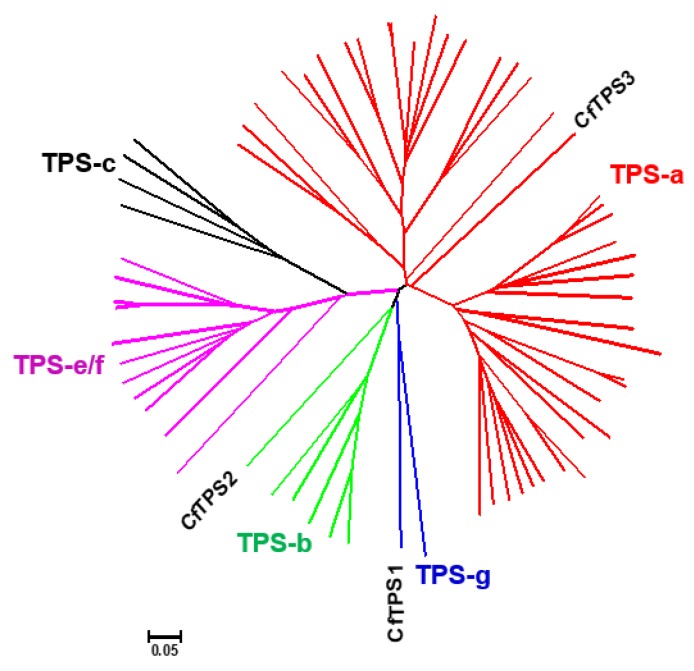
Phylogenetic analysis of three *CfTPS* and selected terpene synthases (*TPSs*) from the dicot *Arabidopsis thaliana* and the monocot *Oryza sativa* based on estimation of pair-wise distances at the amino acid level. Clusters corresponding to five *TPS* subfamilies (*TPS-a, TPS-b, TPS-c, TPS-g, and TPS-f*) are apparent. The genes belonging to the *TPS-a, TPS-b, TPS-c, TPS-g,* and *TPS-e/f* subfamilies were color-coded in red, green, black, blue, and purple, respectively. The Clustal W 2.0 algorithm was used for the alignment. Trees were inferred with the neighbor-joining (NJ) method and *n* = 1000 replicates for bootstrapping.

*CfTPS2* contains a RR(_X_8)W motif, which is conserved among some monoterpene synthases [21] (Figure 4). Bioinformatic analysis using the online ChloroP1.1 (http://www.cbs.dtu.dk/services/ChloroP/) and RaptorX software predicted that both *CfTPS1* and *CfTPS2* contain a transit peptide for chloroplast localization while *CfTPS3* does not (Figure 4). To further clarify the potential roles of the three *CfTPS* proteins, a phylogenetic tree was generated by the neighbor-joining method with 32 TPS sequences from Arabidopsis thaliana and 30 TPSs from Oryza sativa. The results showed that the three *CfTPSs* fell into three TPS subfamilies: *CfTPS1* in the TPS-g subfamily, *CfTPS2* in the TPS-b subfamily, and *CfTPS3* in the TPS-a subfamily (Figure 5).

### 2.5. Catalytic Activities of CfTPSs

To determine the contribution of individual *CfTPS* genes to the biosynthesis of floral terpene volatiles of ‘Kaiser’, we determined the catalytic activities of the proteins encoded by *CfTPS* genes.

**Figure 6:**
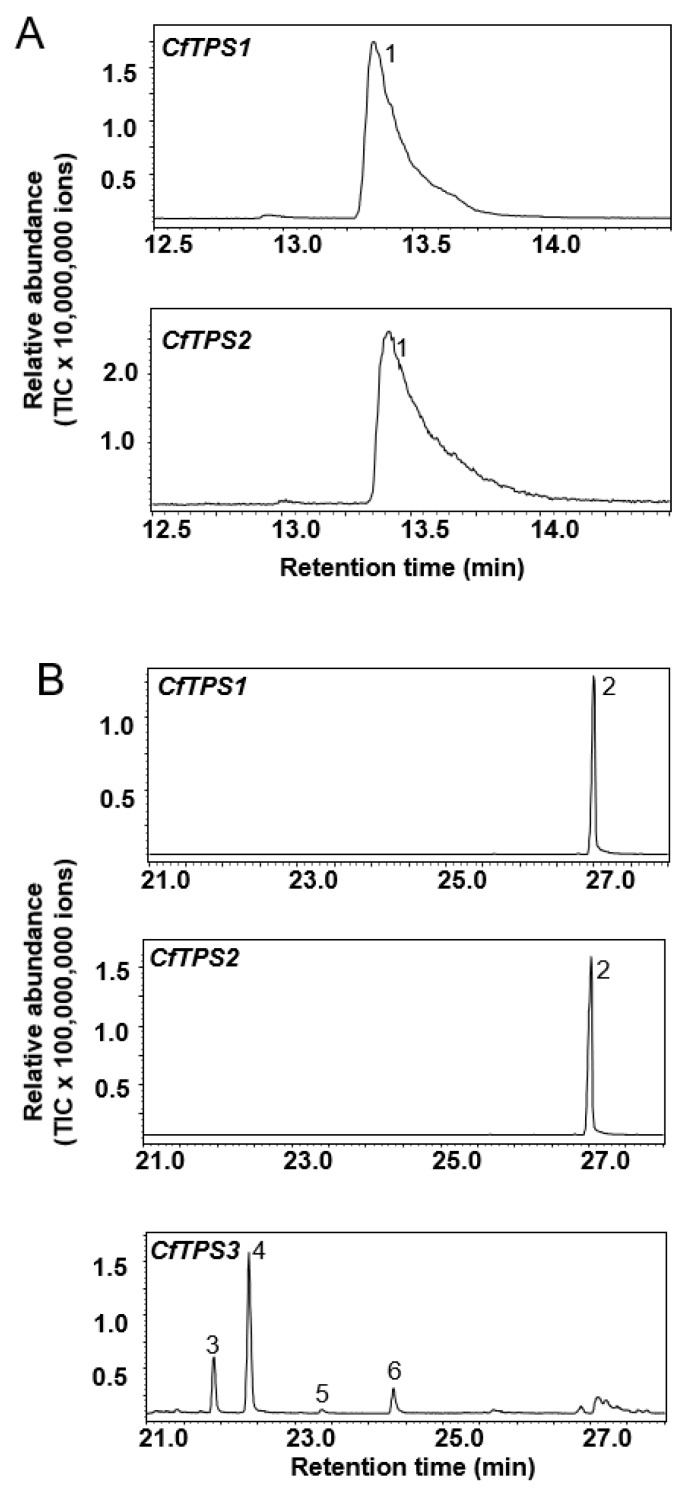
Functional characterization of recombinant *CfTPSs*. (**A**) Enzyme activity of *CfTPS1-2* with geranyl diphosphate as substrate. Linalool (1) was identified as the product of *CfTPS1* and *CfTPS2*. (**B**) Enzyme activity of *CfTPS1-3* with (*E,E*)-farnesyl diphosphate as substrate. Nerolidol (2) was the product of *CfTPS1* and *CfTPS2*. Unknown sesquiterpene (3), α-isocomene (4), (*E*)-β-caryophyllene (5), α-humulene (6) were identified as the main sesquiterpene products of *CfTPS3*.

Full-length cDNA of individual *CfTPS* genes were cloned into a protein expression vector and expressed in *Escherichia coli* to produce recombinant proteins. Then, individual *CfTPS* recombinant proteins were tested in in vitro enzyme assays with GPP and FPP, the substrates of monoterpene synthases and sesquiterpene synthases, respectively. When GPP was used as substrate, both *CfTPS1* and *CfTPS2* produced a single product linalool (Figure 6A). In contrast, *CfTPS3* did not show activity with GPP. When FPP was used a substrate, both *CfTPS1* and *CfTPS2* produced a single product nerolidol, whereas *CfTPS3* catalyzed the formation of multiple sesquiterpenes, including α-isocomene, (*E*)-β-caryophyllene, α-humulene, nerolidol, and an unknown sesquiterpene (Figure 6B).

### 2.6. Expression of CfTPS Genes in Different Floral Organs

**Figure 7:**
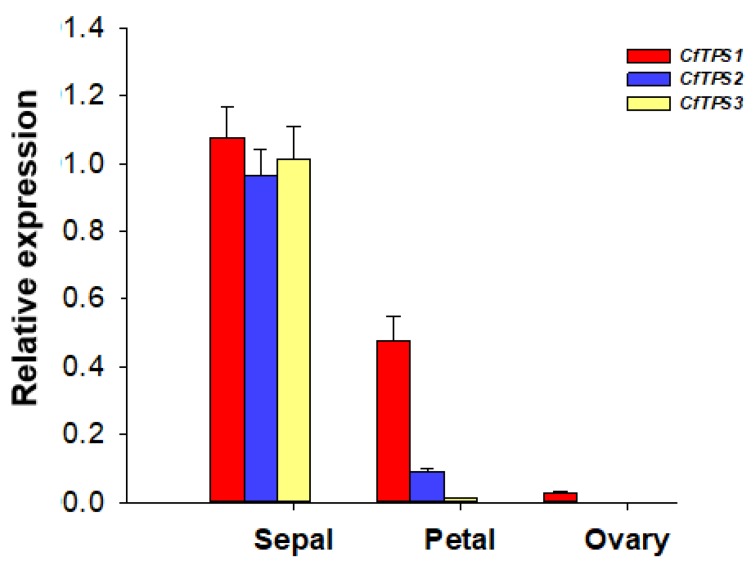
Expression patterns of *CfTPS1-3* in three different organs of *C. florida* ‘Kaiser’ by real-time PCR. Data were presented as means of three biological replicates with error bars indicating standard deviations.

To further assess the role of *CfTPS* genes in floral volatile biosynthesis, the expression of the three *CfTPS* genes in three different floral organs (sepals, transformed-petals, and ovaries) were measured by reverse transcription quantitative PCR (RT-qPCR). All three *CfTPS* genes were expressed in sepals and transformed-petals with higher levels of expression detected in sepals (Figure 7).

## 3. Discussion

In this study, we investigated the chemical composition and biosynthesis of floral scent of ‘Kaiser’, a fragrant cultivar of *C. florida*. The floral scent of ‘Kaiser’ is composed of four main chemical classes: terpenoids, benzenoids, fatty acid derivatives, and nitrogenous compounds (Table 1). This is consistent with the general pattern of chemical composition of floral scents [12,13]. Although only a limited number of terpene compounds (seven monoterpenoids and one sesquiterpene) were detected, they accounted for more than 65% of the total emission (Figure 1). In terms of emission rates of different floral organs, it was somehow surprising that ovaries of ‘Kaiser’ flowers exhibited the highest rates of total emission among the three floral organs examined (Figure 2). Another interesting observation was that the emission rates of individual floral volatiles were different among floral organs. For example, linalool accounted for 74% and 94% of total emissions in sepals and transformed petals, respectively, while limonene was the predominant volatile emitted from ovaries, accounting for 83% of the total emission. It should be noted that although ovaries had the highest rates of volatile emission based on fresh weight of tissues, the contribution of the ovary tissue to the total emission of floral volatile within an intact flower is limited because ovary tissue counts a small percentage of the total fresh weight of a whole flower.

As in other plant species, the biosynthesis of volatile floral terpenes in “Kaiser” is mainly controlled by the expression of key terpene synthase genes. In ‘Kaiser’, *CfTPS1* and *CfTPS2* were found to play an important role in scent terpene biosynthesis. Linalool, the major constituent of ‘Kaiser’ floral scent (Figure 1), was determined as the major product of *CfTPS1* and *CfTPS2* by in vitro enzyme assays (Figure 6). Furthermore, *CfTPS1* and *CfTPS2* showed highest levels of expression in sepals, which is consistent with a strong emission of linalool from these flower organs (Figure 2H).

Although having the same catalytic activity, *CfTPS1* and *CfTPS2* belong to the TPS-g subfamily and TPS-b subfamily, respectively. Enzymes of the TPS-g subfamily have been described to produce mainly acyclic monoterpene alcohols such as linalool [21]. In contrast, members of the TPS-b subfamily usually catalyze the formation of cyclic and acyclic monoterpene hydrocarbons [21]. It is certainly interesting that both *CfTPS1* and *CfTPS2* are involved in making linalool (Figure 6). Linalool was found to be the most abundant floral volatile, representing nearly 60% of the total volatile emission (Figure 1). While the formation of linalool is a conserved function within the TPS-g subfamily, linalool is an unusual product for a TPS-b enzyme. The formation of linalool by *CfTPS2* might be evolved through natural selection from a hydrocarbon-producing TPS-b progenitor. As *CfTPS1* and *CfTPS2*, *CfTPS3* was mainly expressed in sepals (Figure 7). However, its sesquiterpene products could not be detected in the headspace of ’Kaiser’ flowers (Figure 1). It is thus tempting to speculate that the sesquiterpene hydrocarbons produced by *CfTPS3* are rapidly converted into non-volatile terpenoids, presumably through the action of cytochrome P450 monooxygenases. The identification and characterization of such P450 is a worthwhile aim for future studies.

Despite being sterile, the flowers of ‘Kaiser’ are frequently visited by insects. This suggest that the floral volatiles might serve as an olfactory cue for insects. Since ‘Kaiser’ flowers lack a male reproductive part, pollens cannot be a reward for insects. As such, it’s most likely that insects are rewarded by nectar. However, ‘Kaiser’ plants do not benefit from such an interaction due to its inability to produce seeds. Nonetheless, it is sensible to hypothesize that such interactions are mutually beneficial for the fertile relatives from which ‘Kaiser’ was developed. It will be an interesting future research to determine the role of individual constituents for attracting insects in both ‘Kaiser’ and its fertile relatives. Among the floral volatiles of ‘Kaiser’, linalool, the most abundant constituent, has also been identified as the major volatile terpene in flowers of a wide range of plants species from Magnoliaceae to Asteraceae using scent to attract pollinators [24,25,26,27,28,29]. Moreover, linalool contributes to the sweet fragrance noted by humans [23,30,31]. Therefore, linalool will be among the interesting candidate volatiles to be tested. Besides pollinator attraction, terpenes released from flowers may have a role in defenses against florivores [18,32]. Given the sterile property of ‘Kaiser’ flower, it could be speculated that the volatile terpenes emitted from ‘Kaiser’ flowers are probably also involved in defense.

This study provides a justification for a strategy of developing new ornamental cultivars with fragrant sterile flowers. For some species that can be propagated through vegetative tissues, such as through cutting, fruit/seed production is undesired. For such species, new mutant plants could be produced so flowers are sterile but retains the characteristics of scent production.

## 4. Materials and Methods

### 4.1. Plants and Growth

Plants of *Clematis florida* ‘Kaiser’ were grown at the resource nursery in the Institute of Subtropical Crops of Zhejiang Province (120°37′53″ E, 28°0′8″ N), China. All the plants were cultivated under the same conditions of fertilization, irrigation, disease prevention, and pesticide application. The flowers of *C. florida* ‘Kaiser’ were collected for volatile collection and identification at the stage of full blooming in 20–22 April 2018.

### 4.2. Floral Volatile Collection and Identification

Volatiles emitted from intact flowers of *C. florida* ‘Kaiser’ were collected in an open headspace sampling system (Analytical Research Systems, Gainesville, FL, USA) as previously reported with minor modification [33,34]. A single detached inflorescence in a single flask with 150 mL of distilled water were placed in a glass chamber (20 cm diameter, 50 cm tall) covered with a removable lid. Individual floral tissues in *C. florida* ‘Kaiser’ were detached into sepal, petal, and ovary for the volatile collection. Charcoal-purifified air entered the chamber at a flow rate of 1.3 L min^−1^ from the top through a Teflon hose. Volatiles were collected for 4 h by pumping air from the chamber through a SuperQ volatile collection trap (Analytical Research Systems) and eluted using 100 µL methylene chloride containing nonyl acetate (0.003% *w*/*v*) as an internal standard. The semi-quantification relative to the internal standard (peak area) was applied for the quantification of floral volatiles.

Floral volatiles were analyzed by a gas chromatograph (Agilent-9000, Agilent Technologies, Santa Clara, CA, USA) coupled to a quadrupole mass selective detector (Agilent-7000D). Separation was performed on an Agilent HP 5 MS capillary column (30 m × 0.25 mm) with helium as carrier gas (5 ml·min^−1^ of flow rate). A splitless injection (injection temperature 250 °C) was used, and a temperature gradient of 6 °C min^−1^ from 60 °C (3-min hold) to 300 °C was applied. Products were identified using the National Institute of Standards and Technology mass spectral database. Semi-quantification was performed by comparing the peak area of individual compounds to that of the internal standard. Kovat’s retention indices were calculated by injecting the hydrocarbon standard of C_7_ to C_40_ (Sigma-Aldrich, St. Louis, MO, USA) to GC-MS.

### 4.3. RNA Preparation, Transcriptome Sequencing, and Analysis

Total RNA extraction from two biological replicates of full-opened ‘Kaiser’ flowers, transcriptome sequencing, assembling of clean reads, and annotation of transcripts were performed as previously reported [35]. High quality RNA was utilized for library construction, followed by sequencing on the BGISEQ-500 platform (BGI, Wuhan, China). Raw reads were filtered by removing low-quality reads (defined as reads with >20% of the bases with quality scores <10), reads with adaptors, and reads with unknown nucleotides (N bases >5%) by using SOAPnuke to obtain the clean reads. All the clean reads were de novo assembled using Trinity (parameters: minimum assembled contig to report = 150 bp, and min count for K-mers to be assembled by Inchworm = 4), including paired-end joining and gap filling. Following Trinity, the TGI Clustering Tool (TGICL) (parameters: minimum matched length = 35 bp and minimum score = 35) was used to remove redundant sequences and perform further assembly. The assembled reads with more than 70% identity were considered in one cluster. Both consensus cluster sequences and singletons were used to create the unigene dataset. All transcripts were aligned with sequences in the Nucleotide (Nt) database using Blastn, and compared to Non-redundant (Nr), EuKaryotic Orthologous Groups (KOG), Kyoto Encyclopedia of Genes and Genomes (KEGG) and SwissProt databases using Blastx with an e-value < 10^−5^. Gene ontology (GO) terms were retrieved by using Blast2GO software with Nr annotations, and InterProScan5 was used for InterPro annotation with default parameters. Expression levels of unigenes were normalized and calculated as the values of fragments per kilobase of transcripts per million mapped fragments (FPKM) during the assembly and clustering process. Differential expression analysis of unigenes was performed using the DESeq R package (1.10.1). DESeq provides statistical routines for assessing the differential genes expression as differential expressed when the *P*-value < 0.05. The transcriptomic data was deposited in the NCBI Sequence Reads Archive (SRA) with the accession number PRJNA615073.

### 4.4. Identification of CfTPS and Sequence Analysis

To identify putative *TPS* genes from ‘Kaiser’, the flower transcriptome was searched using BlastP algorithm with *TPS* genes from Arabidopsis as queries. Multiple protein sequence alignments were made with Clustal W 2.0. A phylogenetic tree with neighbor-joining (NJ) method was constructed using FastTree 2.1.10 under the JTT t CAT model with 1000 bootstrap replicates. The tree was further edited using MEGA 7.0.21.

### 4.5. Protein Expression and Terpene Synthase Enzyme Assays

TPS enzyme assays was conducted following a procedure previously described [36]. Full-length open reading frames of three *CfTPS* genes were synthesized (Genescript, Nanjing, China) and subcloned into the vector pET32a with an N-terminal his-tag (http://www.emdmillipore.com). To express *CfTPS* proteins, each protein expression construct was transformed into the *E. coli* strain BL21 (DE3) CodonPlus (http://www.agilent.com). An empty pET32a vector without any insert was used as a negative control. After inducing expression of recombinant proteins by isopropyl b-d-1-thiogalactopyranoside (IPTG) for 16 h at 18 °C, the cells were collected by centrifugation and disrupted by 6 × 30 s treatment with an XL2000 probe sonicator (output power 100 W, Misonix, Farmingdale, NY, USA) in ice-chilled extraction buffer. The catalytic activity of *E. coli* expressed recombinant *CfTPS* was performed by using the substrates geranyl diphosphate and (*E,E*)-farnesyl diphosphate. Terpene products were collected by SPME and analyzed by GC-MS. The assays were also performed with crude proteins extracted from *E. coli* expressing the negative control vector, but no terpene products were detected.

### 4.6. Quantitative Real-time PCR of CfTPS Genes in Different Floral Organs

Total RNA preparation was performed as described previously according to the manufacturer’s instructions. First strand cDNA was synthesized from 1 µg total RNA and diluted five-fold for gene expression experiment. The primers (for sequences see Table S3) of target genes for quantitative real-time PCR (qRT-PCR) were selected using primer premier 5.0 software (Premier Biosoft, Palo Alto, CA, USA), and the absence of hairpin structure and primer dimer were predicted by Oligo 6.0 software (Molecular Biology Insights, Colorado Springs, CO, USA). The qRT-PCR experiment was carried out by using a QuantStudio^TM^ 6 Flex Real-Time PCR System (Applied Biosystems, Carlsbad, CA, USA) and SYBR Premix Ex Taq (Takara Biotechnology, Dalian, Liaoning Province, China). The PCR conditions were as follows: 95 °C for 5 min, followed by 40 cycles of 95 °C for 5 s, and 60 °C for 30 s. The qRT-PCR for each sample was repeated three times. The GAPDH gene was used as internal normalizations for different organs. Each primer pair was validated the specificity by melt curve analysis, and the gene expression levels were calculated by the 2XDDCT method. The qRT-PCR results were analyzed by using QuantStudio^TM^ Real-Time PCR Software (Applied Biosystems).

### 4.7. Statistical Analysis

Differences in emission rates of individual terpenoids among different floral organs were analyzed by one-way analysis of variance (ANOVA). The analyses were conducted with SAS V8 software (Version 8.02. SAS Institute, Cary, NC, USA) and all statistical effects were considered significant at *P* < 0.05. All *P*-value for multiple comparisons have been corrected by Bonferroni correction.

## Figures and Tables

**Table 1:** The major volatile compounds identified in the full flowers of *Clematis florida* ‘Kaiser’.

No.	Compounds	Retention Index		Emission Rate (ng/g/h) ^c^
		Calc ^a^	Lit ^b^	
**Terpenoids**				
1	Limonene	1033	1031	28.46 ± 12.62
2	*trans*-Linalool oxide	1053	1081	3.41 ± 0.64
3	3-Carene	1079	1017	2.96 ± 0.30
4	5-Caranol	1095	1125	4.12 ± 0.53
5	Linalool	1107	1101	423.22 ± 33.71
6	6-Ethenyldihydro-2,2,6-trimethyl-2*H*-Pyran-3(4*H*)-one	1115	1108	4.87 ± 0.64
7	Nerolidol	1572	1564	10.11 ± 0.74
**Benzenoids**				
8	Benzyl alcohol	1038	1036	2.62 ± 0.22
9	2-Phenylacetaldehyde	1049	1047	52.43 ± 7.49
10	Phenylethyl alcohol	1119	1116	37.45 ± 5.49
11	1,4-Dimethoxybenzene	1170	1158	12.73 ± 1.87
12	2-(2-Butoxyethoxy)ethanol	1195	1192	15.36 ± 2.47
13	2-Phenoxyethanol	1226	1225	48.69 ± 11.24
14	2-Phenylethylacetate	1265	1258	26.97 ± 4.12
15	1,3,5-Trimethoxybenzene	1417	1392	3.75 ± 0.37
**Fatty acid derivatives**				
16	2-Hydroxyethyl methacrylate	1019	985	1.76 ± 0.26
17	Nonanal	1110	1104	2.70 ± 0.33
18	Decanal	1212	1206	5.24 ± 0.75
19	Tetradecane	1406	1400	2.39 ± 0.41
20	2,6,10,15-Tetramethylheptadecane	1506	1660	4.87 ± 0.71
**Nitrogen-containing compounds**				
21	Benzyl cyanide	1146	1144	7.49 ± 1.12
22	Phenylacetaldoxime	1253	1402	1.32 ± 0.25
23	2-Phenylnitroethane	1311	1283	10.32 ± 1.86

Note: ^a^ Separation of volatile compounds was performed on an Agilent HP 5 MS capillary column. The retention indices were gained by injecting the C_7_ to C_40_ hydrocarbon standard (Sigma-Aldrich, St Louis, MO, USA) to GC–MS. ^b^ Retention indices archived by Adams (2001). ^c^ Emission rate was calculated as ng/g fresh weight/h using semi-quantification.

## Supplementary Materials

The following are available online. Figure S1. The length distribution of assembled unigene from *C. florida* ‘Kaiser’; Figure S2. KEGG annotation and GO classification of putative unigenes in *C. florida* ‘Kaiser’. Table S1. Summary of the *Clematis florida* ‘Kaiser’ transcriptome. Table S2. Statistics of annotation on unigenes against public databases. Table S3. The primers of *CfTPS* used for qRT-PCR.
