# Supplementary material for: Composition and Biosynthesis of Scent Compounds from Sterile Flowers of an Ornamental Plant Clematis florida cv. ‘Kaiser’
Source: Molecules. 2020 Apr 8;25(7):1711. doi: 10.3390/molecules25071711 (PMC7180759; doi:10.3390/molecules25071711)
Supplement: Supplementary file 1 [file molecules-25-01711-s001.pdf]

*Article*

# Composition and Biosynthesis of Scent Compounds from Sterile Flowers of an Ornamental Plant *Clematis florida* cv. 'Kaiser'

**Yifan Jiang[1#*], Renjuan Qian[2#], Wanbo Zhang[1], Guo Wei[3], Xiaohua Ma[2], Jian Zheng[2], Tobias G. Köllner[4] and Feng Chen[1,3*]**

**Supplementary Figures**

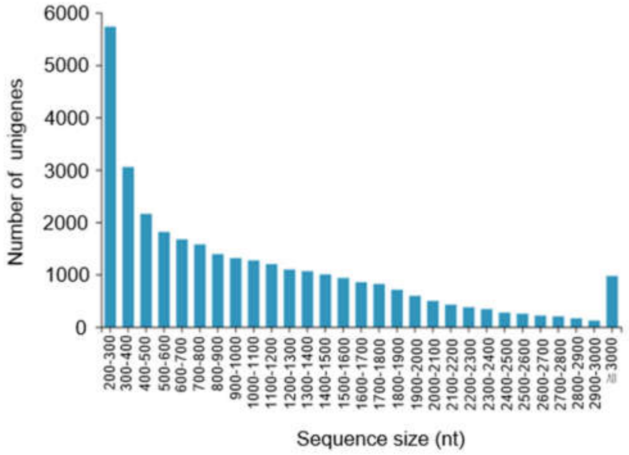


**Figure 1.** The length distribution of assembled unigenes from *C. florida* 'Kaiser'. The horizontal and vertical axis showed the size and the number of transcripts, respectively.

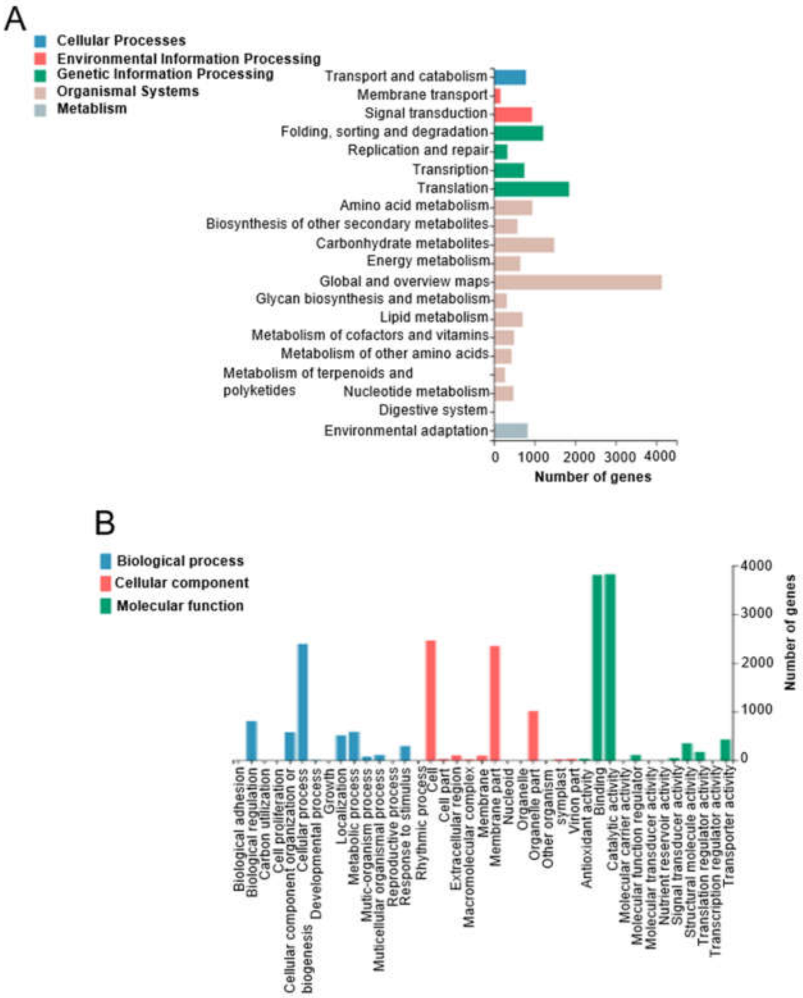


**Figure S2.** (**A**) KEGG annotation of putative proteins. The y-axis indicates the name of the KEGG metabolic pathway. The x-axis indicates the percentage of the number of unigenes annotated to the pathway out of the total number of unigenes annotated. The unigenes were divided into five branches according to the KEGG metabolic pathway: Cellular Processes, Environmental Information Processing, Genetic Information Processing, Metabolism and Organismal Systems. (**B**) GO classification of unigenes in *C. florida* 'Kaiser'. Gene Ontology terms are classified into three main categories: biological process (BP), cellular component (CC) and molecular function (MF).

## Supplementary Tables

**Table S1.** The summary of the *Clematis florida* 'Kaiser' transcriptome.

| Transcript | Unigene |
|---|---|
| Total assembled bases | 22038123 |
| Total number of genes | 32048 |
| Max length of unigenes (bp) | 11031 |
| Min length of unigenes (bp) | 297 |
| N50 (bp) | 1323 |
| GC percentage (%) | 44.68% |

**Table S2.** The statistics of annotation on unigenes against public databases.

| Database | Number of Annotated Unigenes | Percentage of Annotated Unigenes (%) |
|---|---|---|
| Nr | 24939 | 77.82 |
| Swiss-prot | 18484 | 57.68 |
| KOG | 19429 | 60.62 |
| KEGG | 18981 | 59.23 |
| GO | 16150 | 50.39 |
| Total | 32048 | 100 |

**Table S3.** Primers of *Cf*TPS used for qRT-PCR.

| Gene No | Forward | Reverse |
|---|---|---|
| *Cf*TPS1 | CAGTCTTCAACGCCTATCCATT | TGTTATCGCCAGCATCATCATT |
| *Cf*TPS2 | AGAATCCGTTCAGCGACTAGA | GCGTCATAGGAGCCATAACATT |
| *Cf*TPS3 | CTTGGCGTTGGCTATCACTT | CCGTTCTCATCTCTGTACTTGTTG |
| *Cf*GAPDH | AACCCTGAGGAGATTCCA | CACCACCCTTCAAGTGAGCAG |
